# 2520: Participant recruitment program at the University of Michigan CTSA

**DOI:** 10.1017/cts.2017.288

**Published:** 2018-05-10

**Authors:** Meghan Spiroff, Lisa Connally, Anita Johnson, Aalap Doshi, Patricia Piechowski

**Affiliations:** University of Michigan School of Medicine, Ann Arbor, MI, USA

## Abstract

OBJECTIVES/SPECIFIC AIMS: Across the Clinical and Translational Science Award (CTSA) Consortium, participant recruitment into clinical trials is essential to advance science. Without proper participant recruitment, clinical trials do not result in gains in scientific knowledge, wastes time, funds, and other resources (Mahon *et al.*, 2015). METHODS/STUDY POPULATION: Participant recruitment programs across the consortium are inconsistent in staffing, program services, and program goals. The participant recruitment program at the University of Michigan’s (U-M) Michigan Institute for Clinical & Health Research (MICHR) provides expertise, tools, and resources to facilitate participant recruitment in clinical and health research studies. RESULTS/ANTICIPATED RESULTS: We will explain our program infrastructure, staffing, services, and discuss how we maintain an engaged registry with over 27,000 participants interested in research studies at U-M. DISCUSSION/SIGNIFICANCE OF IMPACT: Proper recruitment into clinical trials results in findings that are relevant for genetic, cultural, linguistic, racial/ethnic, gender, and age differences (Cottler *et al.*, 2013). We hope to share our best practices that aid in the development and success of participant recruitment across the CTSA Consortium.

